# Ocular Manifestations of Pediatric Rhinosinusitis: A Comprehensive Review

**DOI:** 10.3390/diseases12100239

**Published:** 2024-10-02

**Authors:** Antonino Maniaci, Caterina Gagliano, Salvatore Lavalle, Nicolien van der Poel, Luigi La Via, Antonio Longo, Andrea Russo, Marco Zeppieri

**Affiliations:** 1 Department of Medicine and Surgery, University of Enna “Kore”, Piazza dell’Università, 94100 Enna, Italy; antonino.maniaci@unikore.it (A.M.);; 2 Deparment of Eye Clinic, Catania University San Marco Hospital, Viale Carlo Azeglio Ciampi, 95121 Catania, Italy; 3 Department of Otorhinolaryngology, Antwerp University Hospital, 2000 Antwerp, Belgium; 4 Faculty of Medicine and Translational Neurosciences, University of Antwerp, 2000 Antwerp, Belgium; 5 Department of Anesthesia and Intensive Care, Azienda Ospedaliero Universitaria Policlinico “G. Rodolico-San Marco”, 95123 Catania, Italy; 6 Department of Ophthalmology, University of Catania, 95123 Catania, Italy; 7 Department of Ophthalmology, University Hospital of Udine, 33100 Udine, Italy

**Keywords:** rhinosinusitis, pediatrics, ocular complications

## Abstract

*Background:* Rhinosinusitis is a common childhood illness that may result in a number of ocular problems. The goal of this thorough analysis is to compile the available data regarding the kinds, prevalence, and treatment of ocular problems related to juvenile rhinosinusitis. *Methods:* A comprehensive analysis of electronic databases, such as PubMed, Embase, and Cochrane Library, was carried out to pinpoint pertinent research articles written in English and published between the beginning and August of 2023. Included were studies that discuss ocular problems in young children suffering from rhinosinusitis. Study characteristics, ocular complication kinds, occurrence rates, and management approaches were the main topics of data extraction. *Results*: A significant number of pertinent research on ocular problems related to juvenile rhinosinusitis was found through the literature search. Preliminary searches indicated that orbital cellulitis, subperiosteal abscess, cavernous sinus thrombosis, and optic neuritis are probably the most often reported ocular problems. It is expected that these problems will occur at a wide range of incidence rates, from somewhat unusual to more prevalent. Depending on the severity of the consequences, management techniques are expected to require a combination of surgical and medicinal procedures. Healthcare professionals will benefit from the findings since they will improve knowledge of the clinical presentation, diagnosis, and treatment of these potentially blinding consequences. The evaluation also assists in identifying knowledge gaps and guides future directions for this field of study, both of which are critical to enhancing patient outcomes. *Conclusions:* The wide range of topics included in this study will help to better understand the burden of ocular consequences related to juvenile rhinosinusitis and will make it easier to build preventative and treatment plans that work better.

## 1. Introduction

Pediatric rhinosinusitis is a prevalent and very serious disease, affecting up to 4% of children annually [1]. Despite being largely characterized by inflammation of the nose and sinuses, this sickness can cause serious ocular consequences that could endanger both vision and general health [2,3,4]. Children are more vulnerable to these issues due to their unique anatomical and immunological characteristics, such as the sinuses’ close proximity to the orbit and their developing immune systems [3,4,5]. Pediatric rhinosinusitis can present with a wide variety of ocular symptoms, from moderate conjunctivitis to severe diseases such as orbital cellulitis, subperiosteal abscess, and intracranial extension [4,6,7]. Proptosis, chemosis, ophthalmoplegia, and, in extreme situations, irreversible blindness or potentially fatal disorders, such as cavernous sinus thrombosis, can be the outcomes of these problems [4,7,8,9]. The seriousness of these consequences highlights how crucial it is to identify problems early and take the right action to treat them. Diagnosing ocular problems in children with rhinosinusitis, however, is a difficult task. It is necessary to have a high index of clinical suspicion when dealing with symptoms that are confusing or overlap with other illnesses [10]. To confirm the diagnosis and determine the degree of involvement, advanced imaging methods including magnetic resonance imaging (MRI) and computed tomography (CT) are frequently needed [11]. Systemic antibiotics and thorough observation are the usual treatment techniques; surgery is only used in cases with ocular abscess or intracranial dissemination [12]. Notwithstanding the clinical importance of ocular consequences in juvenile rhinosinusitis, there is a dearth of thorough studies that comprehensively summarize the current body of information in this area. Our knowledge of the entire range of these difficulties is lacking because prior assessments have a tendency to concentrate on particular elements or management techniques [13,14]. In order to close this knowledge gap, a complete analysis of the literature on the ocular symptoms of pediatric rhinosinusitis is provided in this review. To be more precise, our goals are to 1. describe the kinds and frequency of ocular complications in pediatric rhinosinusitis; 2. determine risk factors linked to the emergence of these complications; 3. assess the effectiveness of current diagnostic techniques; 4. evaluate treatment modalities and their results; and 5. point out areas that need more research in order to enhance patient care. Our goal in compiling these data is to provide medical practitioners with the tools they need to identify and treat these issues in a timely manner, which will eventually lead to better patient outcomes. In addition, the results of this analysis may help direct future investigations and make policy decisions on the distribution of funds for the detection, management, and avoidance of ocular problems in pediatric rhinosinusitis. The pediatric population was defined for the purposes of this review as those who are between the ages of 0 and 18. This age range covers the window of time during which immunological and structural characteristics may predispose individuals to ocular consequences of rhinosinusitis and corresponds with the recommendations of major pediatric organizations. We emphasized in our research any differences we saw between younger and older children and adolescents in this age range. Our aim is to improve clinical practice, research focus, and policy formation in this crucial area of pediatric health by conducting a thorough analysis of the ocular symptoms of pediatric rhinosinusitis.

## 2. Materials and Methods

### 2.1. Search Strategy and Eligibility Criteria

This comprehensive review employed a systematic and comprehensive search approach to ensure that the body of literature on the ocular symptoms of pediatric rhinosinusitis is fully captured. Utilizing a variety of pertinent electronic databases, such as PubMed, Embase, Scopus, Web of Science, and the Cochrane Library, the review team performed an extensive literature search. A combination of keywords and MeSH phrases pertaining to pediatric rhinosinusitis, ocular symptoms, and particular complications such as intracranial problems, subperiosteal abscess, preseptal cellulitis, and orbital cellulitis was included in the search. In order to guarantee that the most recent evidence is presented, the search covered articles published between each database’s creation date and the present. However, the search only included publications published in English in order to make the review process more feasible, which may omit some potentially important studies. To keep the focus on the most pertinent and trustworthy sources of information, the qualifying requirements for this comprehensive assessment were spelled out in detail. The following are the inclusion criteria for this research: original research articles, such as observational studies, case series, and case reports; studies reporting ocular manifestations or complications related to pediatric rhinosinusitis; and systematic reviews and meta-analyses addressing ocular manifestations in pediatric rhinosinusitis. Pediatric patients with rhinosinusitis, aged 0–18 years, were the focus of the investigations. In order to distinguish any variations between younger children and older adolescents up to the age of 18, we tried to stratify the data by age where possible, even though the primary analysis covered the entire 0–18 age range. Studies that only included adult patients, studies that did not report ocular signs or consequences, opinion pieces, editorials, narrative reviews, conference abstracts and proceedings, and editorials were the exclusion criteria. By using these criteria, it was possible to make sure that the included studies offered a thorough and trustworthy summary of the most recent data about the ocular manifestations of pediatric rhinosinusitis.

### 2.2. Study Selection and Data Extraction

In order to reduce bias and guarantee the validity of the included research, a strict two-step procedure was used in the study selection process for this comprehensive review. The titles and abstracts of the retrieved papers were screened by two impartial reviewers in accordance with the eligibility requirements. After that, the same two reviewers evaluated each article in full to decide whether or not to include it. Any differences of opinion among the reviewers were settled by consensus and debate, or if required, by bringing in a third reviewer. By ensuring that the study selection procedure was methodical, transparent, and repeatable, this strategy contributed to improving the comprehensive review’s overall quality and dependability. The procedures described for this review are intended to guarantee consistency, completeness, and dependability in the data collection process. Data extraction is an essential phase in the comprehensive review process. A uniform data extraction form was created to gather pertinent data from the included studies. These data will encompass a broad range of items, including study characteristics (authors, year, design, and country), patient characteristics (age, gender, and comorbidities), ocular manifestations and complications, management strategies and results, and psychosocial implications. Data from the included studies were extracted independently by two reviewers; disagreements were settled by discussion or the involvement of a third reviewer. By reducing bias and mistakes in the data extraction process, this method guaranteed that the information gathered is accurate, comprehensive, and trustworthy.

### 2.3. Data Synthesis

The purpose of the data synthesis techniques used in this comprehensive review is to offer a thorough and insightful summary of the available data about the ocular manifestations of pediatric rhinosinusitis. The data extraction process was subjected to both a thematic and descriptive analysis. The descriptive analysis aimed to provide a comprehensive review of the existing information by summarizing the important results regarding ocular symptoms in pediatric rhinosinusitis. A fuller exploration of particular features, including the prevalence, clinical presentation, diagnostic method, management techniques, results, and psychosocial consequences of ocular symptoms, was provided by the thematic synthesis. By assisting in the identification of patterns, trends, and gaps in the current literature, this method offered insightful information for next studies and therapeutic applications. Furthermore, based on the synthesis of information, the comprehensive review identifies knowledge gaps and opportunities for future research, which will serve to lead future studies in this crucial field.

## 3. Results

Considerable insights into the ocular symptoms of pediatric rhinosinusitis were revealed by a comprehensive analysis of the literature conducted over a period of almost five decades. As represented in Table 1, over 5700 individuals aged 0–18 years were taken into account, which consistently found preseptal and orbital cellulitis as the main ocular symptoms (Table 1).

The prevalence of these conditions varied significantly among the studies. The incidence of preseptal cellulitis was notably high, as indicated by several investigations that documented its presence in all cases investigated. In particular, children under the age of 5 are identified as a major risk factor for ocular problems. This observation implies that younger children may have a higher susceptibility to the transmission of sinus infections to neighboring orbital tissues. Additional recognized risk factors, such as ethmoid sinusitis and increased duration of symptoms, emphasize the need of timely identification and treatment. Diagnostic methodologies have developed throughout time, with CT scans becoming the benchmark, employed in almost all examined research. The integration of MRI in recent research suggests a movement toward more sophisticated imaging methods, which may provide enhanced resolution of soft tissues and limit radiation exposure. The management approaches uniformly included antibiotic therapy, which underscores its vital significance in the treatment of these illnesses. Nevertheless, the significant disparity in surgical intervention rates (ranging from 1% to 100%) indicates a dearth of uniform standards for surgical treatment. This discrepancy could be attributed to variations in the severity of the disease, local customs, or changing treatment paradigms over the measured decades. Importantly, the results were consistently positive in all the investigations, with the majority showing recovery rates exceeding 95%. The lower recovery rate shown in the first trial in comparison to more recent ones could perhaps be attributed to the progress made in medical treatment and the implementation of earlier intervention techniques across time. The present analysis highlights the significance of promptly identifying and effectively treating ocular consequences in cases of pediatric rhinosinusitis. Although treatment results have shown improvement over time, the ongoing presence of these consequences emphasizes the necessity for ongoing awareness and study in this field.

### 3.1. What Are the Most Commonly Reported Ocular Manifestations Associated with Pediatric Rhinosinusitis and What Is Their Prevalence?

The degree and appearance of pediatric rhinosinusitis’s ocular symptoms can differ. Presectal cellulitis, ocular cellulitis, subperiosteal abscess, and intracranial extension are the most frequently reported sequelae [18]. Preseptal cellulitis is the most common ocular complication among children with orbital complications of rhinosinusitis. It is characterized by inflammation and infection of the eyelid and periorbital soft tissues, and its frequency ranges from 60% to 80% [20,21]. With a reported prevalence of 15% to 30%, orbital cellulitis, which involves infection of the orbital contents posterior to the orbital septum, is the second most common complication [22]. Between 5% and 15% of cases result in the establishment of a subperiosteal abscess, which is a collection of purulent material between the periorbita and the bony orbit [23]. The most serious consequence, intracranial extension, is uncommon but can manifest as meningitis, subdural or epidural abscess, or cavernous sinus thrombosis [24]. It is expected that between 1% and 3% of children with rhinosinusitis will experience intracranial problems [25]. For healthcare professionals to recognize and treat ocular manifestations promptly, they must have a thorough understanding of their prevalence and range.

### 3.2. Are There Any Specific Risk Factors or Predictors for Developing Ocular Complications in Children with Rhinosinusitis?

For the purpose of early detection and treatment, it is essential to identify risk factors and predictors for the emergence of ocular problems in children with rhinosinusitis. Numerous research endeavors have examined plausible variables that could potentially augment the probability of orbital involvement in pediatric patients. The patient’s age is a major risk factor, with younger children more vulnerable to orbital problems [26]. The growing immune system, the sinuses’ physical closeness to the orbit, and the existence of more permeable and thinner bone barriers dividing these structures could all be contributing factors to this higher risk in younger patients [27]. The amount of time that rhinosinusitis symptoms last before a person seeks medical assistance is another significant risk factor. Individuals who have untreated or persistent sinusitis are more likely to have difficulties in their eyes [28]. The infection can grow and possibly infiltrate the orbital tissues as a result of treatment delay. Furthermore, the likelihood of orbital problems may be impacted by the existence of particular sinuses implicated in the infectious process. Because the ethmoid air cells are so close to the orbit, ethmoid sinusitis has been linked to an increased risk of orbital involvement, especially in younger children [29]. The development of ocular problems is also influenced by the microbiology of the underlying rhinosinusitis. An increased risk of orbital extension has been associated with infections brought on by specific bacterial pathogens, including anaerobic bacteria, Staphylococcus aureus, and Streptococcus species [30,31]. These organisms have virulence factors, which make it easier for them to invade tissue and can result in more severe symptoms. Moreover, a higher risk of ocular problems has been linked to the occurrence of polymicrobial infections, which involve numerous bacterial species [32]. In the context of rhinosinusitis, certain comorbidities and underlying medical disorders may potentially put children at risk for developing ocular consequences. Orbital involvement is more common in patients in immunocompromised states, such as those receiving chemotherapy or having congenital immunodeficiencies [33]. Furthermore, some children have pre-existing anatomical conditions that could transmit infection, including congenital dehiscences or fractures in the bone walls that divide the orbit from the sinuses [34]. The development of ocular problems in pediatric rhinosinusitis has also been linked to socioeconomic factors and inequities in healthcare access. According to studies, children from low-income households and those with little access to medical treatment are more likely to have orbital involvement in advanced stages when they first appear [35,36]. Infection progression and the emergence of more serious consequences might be facilitated by delayed diagnosis and treatment, which is frequently brought on by financial hardships or a shortage of healthcare resources. In addition to these risk factors, certain clinical manifestations of rhinosinusitis in children may further indicate the likelihood of ocular sequelae. Suspicion for orbital involvement should be raised in the presence of periorbital edema, erythema, and discomfort, particularly when these symptoms are coupled with systemic signs such as fever and leukocytosis [37]. More specific indicators of orbital cellulitis or subperiosteal abscess include proptosis, ophthalmoplegia, and visual abnormalities [17]. Healthcare professionals must be aware of these risk factors and predictors in order to keep a close eye on children with rhinosinusitis and rapidly identify any ocular problems. Clinicians can stratify patients according to their risk profile and start the right diagnostic and therapeutic treatments by taking into account criteria including age, duration of symptoms, affected sinuses, microbiology, comorbidities, and clinical presentation. In the end, this risk stratification strategy can improve patient outcomes and lower the incidence of long-term sequelae by assisting in the early detection and management of ocular issues.

### 3.3. How Do the Types and Severity of Ocular Manifestations Vary Based on the Age of the Pediatric Patient or the Duration of Rhinosinusitis?

Depending on the patient’s age and the length of the underlying sinus infection, there can be differences in the kinds and severity of ocular symptoms in juvenile rhinosinusitis. Compared to older children and adolescents, younger children—especially those under the age of five—are more likely to experience serious orbital problems [38]. Younger patients are more susceptible because of their developing immune systems, the lack of fully formed bone barriers separating the sinuses from the orbit, and the higher incidence of ethmoid sinusitis in this age range [3]. Regarding the particular forms of ocular symptoms, older children and adolescents are more likely to experience preseptal cellulitis, whereas younger children are more likely to experience orbital cellulitis and subperiosteal abscess [9]. The anatomical development of the sinuses and the infection’s propensity to move more easily to the orbital tissues in younger patients may be the causes of this variation in presentation. Furthermore, younger children are more prone to experience intracranial problems, even if they are uncommon, because of their underdeveloped immune systems and the possibility of an infection progressing quickly [39]. The length of rhinosinusitis prior to the development of ocular symptoms is another important factor influencing how serious orbital problems can become. Individuals who have untreated or protracted sinusitis are more likely to have orbital involvement in more severe stages [28]. If the infection is not promptly diagnosed and treated, it can progress and inflict more extensive harm to the orbital tissues. Research indicates that individuals with subperiosteal abscess or orbital cellulitis frequently experienced antecedent sinusitis for a longer period of time than those with preseptal cellulitis [40]. Furthermore, the length of rhinosinusitis might affect the infection’s microbiology and, in turn, the intensity of its ocular symptoms. Resistant bacterial strains or polymicrobial flora are more likely to produce prolonged or recurring sinus infections, which might result in more aggressive and challenging-to-treat orbital sequelae [31]. Anaerobic bacteria have been related to a higher prevalence of subperiosteal abscess formation and the requirement for surgical intervention; they are frequently connected with chronic sinusitis [32]. The interaction between the patient’s age and the length of rhinosinusitis might also affect how severe the ocular symptoms are. The risk of developing serious ocular sequelae, such as intracranial extension or orbital cellulitis with subperiosteal abscess, is greater in younger children who have untreated or protracted sinusitis [41]. This confluence of elements produces an ideal environment for the infection to spread quickly and the emergence of consequences that could endanger life or sight. It is imperative for healthcare practitioners to comprehend the impact of age and duration of rhinosinusitis on the nature and intensity of ocular symptoms in order to swiftly identify and treat these issues in pediatric patients. Younger children should be constantly monitored and treated aggressively to prevent the development of severe ocular problems, especially if they have persistent symptoms of sinusitis. In these susceptible patients, early diagnosis, adequate antibiotic medication, and surgical intervention when necessary can greatly improve results and lower the risk of long-term consequences [7].

### 3.4. What Are the Most Effective Diagnostic Tools and Imaging Modalities for Identifying and Assessing Ocular Complications in Children with Rhinosinusitis?

**Figure 1:**
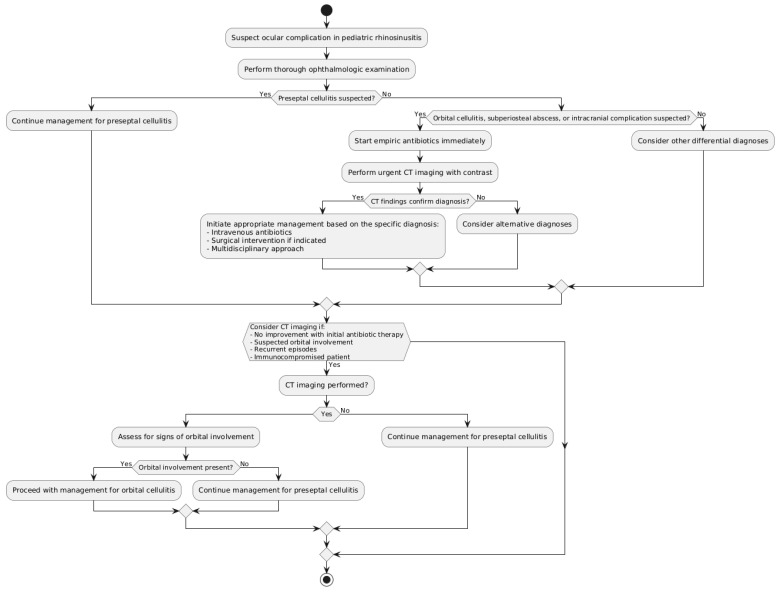
Most effective diagnostic tools and imaging modalities for ocular complications.

For children with rhinosinusitis, a timely and precise identification of ocular problems is essential to start the right course of treatment and avoid potentially fatal or severely disfiguring outcomes. The identification and evaluation of orbital involvement need a mix of imaging modalities and clinical examination. Computed tomography (CT) scanning, magnetic resonance imaging (MRI), and a comprehensive ophthalmological evaluation are the most efficient diagnostic methods and imaging modalities [42,43]. When assessing a child who may have ocular problems from rhinosinusitis, a thorough ophthalmological examination is the initial step (Figure 1).

Fundoscopy, ocular motility testing, pupillary reflex evaluation, and visual acuity testing should all be included in this evaluation [43]. Suspicion of orbital cellulitis or subperiosteal abscess is increased in the presence of proptosis, chemosis, ophthalmoplegia, or impaired visual acuity. The degree of ocular involvement can be ascertained by comparing the results between the affected and unaffected eyes [11]. The preferred imaging technique for identifying and evaluating ocular problems in pediatric rhinosinusitis is computed tomography (CT) scanning [5]. CT allows for the diagnosis of sinus opacification, mucosal thickening, and orbital inflammation by providing precise image of the sinuses, orbit, and surrounding soft tissues. Contrast-enhanced CT scans can identify subperiosteal or intraorbital abscesses, distinguish between preseptal and post-septal involvement, and further characterize the extent of the infection [8]. When intervention is required, the use of multiplanar reconstructions using fine-cut, high-resolution CT images has enhanced diagnostic accuracy and made surgical planning easier [16]. Another useful method for assessing ocular problems is magnetic resonance imaging (MRI), especially in cases where cerebral extension is suspected or when CT results are unclear [44]. Better in defining the amount of ocular inflammation, abscess formation, and cerebral involvement, MRI offers greater soft-tissue contrast. Diffusion-weighted imaging (DWI) sequences can be used to distinguish between subperiosteal abscesses and orbital cellulitis because the latter usually exhibits restricted diffusion [45]. When radiation exposure from CT scans is a problem, MRI is also helpful [46]. This is particularly true for young children who may need recurrent imaging. Apart from these major diagnostic techniques, other instruments can offer significant insights into the evaluation of ocular problems. When used by a skilled practitioner, ultrasonography can be a rapid and radiation-free way to assess orbital inflammation and find subperiosteal collections [45,46,47]. Treatment decisions can be guided by ophthalmological examinations, such as color vision assessment and visual field testing, which can help ascertain the functional consequences of the ocular involvement [47]. The patient’s age, suspected severity of the ocular problems, and clinical presentation should all be taken into consideration when selecting imaging modalities and diagnostic instruments. For the majority of cases, a comprehensive ophthalmological examination along with contrast-enhanced CT scanning is enough for preliminary assessment and treatment planning. If the CT results are equivocal or there is a suspicion of cerebral extension, an MRI may be deferred [48,49,50,51,52,53,54]. When these diagnostic techniques and imaging modalities are used effectively, it becomes possible to accurately identify and assess the ocular issues that children with rhinosinusitis may have. Initiating prompt and adequate treatment is crucial for early recognition and characterization of orbital involvement, as this reduces the risk of irreversible visual impairment or potentially fatal consequences [9]. Several investigations have highlighted the difficulty in acquiring sufficient imaging in very young pediatric patients, mostly attributed to their inability to maintain stillness during CT or MRI acquisitions [55,56,57,58,59,60]. Although sedation rates were not specifically documented in any trials, it was suggested that patients under around 4–5 years of age would require general anesthetic to effectively undergo advanced imaging. Particular emphasis was placed on careful evaluation of radiation exposure from CT scans compared to the usefulness of MRI, especially when regular follow-up imaging was necessary to track illness development.

### 3.5. What Are the Current Treatment Approaches (Medical and Surgical) for Managing Different Types of Ocular Manifestations in Pediatric Rhinosinusitis and How Do They Compare in Terms of Outcomes?

Depending on the nature and extent of the problem, a combination of medication and surgical techniques is used to treat the ocular symptoms of pediatric rhinosinusitis [61,62,63]. Eliminating the infection, lowering inflammation, and halting vision loss or cerebral spread are the main objectives of treatment [64,65]. In order to achieve the best results, suitable therapy must be started as soon as possible [7]. For the majority of instances of preseptal and early-stage orbital cellulitis, medical therapy is the initial line of treatment [66]. In order to treat both aerobic and anaerobic organisms, broad-spectrum intravenous antibiotics are usually administered [49]. An example of this would be a third-generation cephalosporin combined with either metronidazole or clindamycin. To enhance sinus outflow and lessen inflammation, topical nasal steroids and nasal decongestants can also be applied [50]. To evaluate the efficacy of medical treatment and identify any infection progression, repeated ophthalmological tests and close monitoring of the patient’s clinical response are crucial [51]. Surgery is frequently required in addition to medicinal therapy in situations of severe orbital cellulitis or subperiosteal abscess [23]. The size and location of the abscess, the patient’s clinical status, and the patient’s reaction to the first round of medical treatment all play a role in the decision to proceed with surgery [52]. Surgical treatments for orbital abscess drainage via an exterior or transnasal route and endoscopic sinus surgery to empty the afflicted sinuses are available [53]. The accuracy and safety of these surgical operations have increased thanks to image-guided navigation devices, especially for young patients whose anatomy is still growing [54]. The volume of the abscess, the patient’s age, and the existence of visual abnormalities all influence the decision between medicinal and surgical subperiosteal abscess treatment [55]. Research indicates that for children under 9 years old, medical therapy alone may be enough for tiny abscesses (<0.5–1 mL); nevertheless, immediate surgical drainage is necessary for bigger abscesses or those that cause vision impairment [56]. Nonetheless, to guarantee abscess clearance and avoid recurrence, serial imaging and vigilant observation are required [9]. In terms of results, it has been demonstrated that treating the ocular consequences of pediatric rhinosinusitis can be accomplished using both medicinal and surgical methods. When treating preseptal and early orbital cellulitis, medical care has a good success rate, and patients typically exhibit clinical improvement 24 to 48 h after starting antibiotic medication [57]. Numerous studies have documented the use of saline nasal sprays or rinses as supplementary treatment in conjunction with antibiotics and other medical interventions. Nasal saline is purported to facilitate the removal of infectious material and mucus from the nasal cavities and sinuses. This intervention was advised when the pediatric patients were able to tolerate it, in order to facilitate sinus drainage and the administration of topical therapy. Nevertheless, none of the research measured the magnitude of saline usage or presented comparison statistics on the results achieved with and without saline therapy. When compared to medical therapy alone, surgical intervention has been linked to quicker symptom clearance, shorter hospital stays, and decreased incidence of complications [58]. However, the exact form of ocular manifestation and the existence of underlying risk factors may affect the best course of action and its results. For instance, longer antifungal medication and more vigorous surgical debridement may be necessary for patients with fungal infections or cerebral expansion [59]. Children who are immunocompromised or who have experienced recurrent sinusitis in the past may also be more susceptible to treatment failure and need to be closely monitored [44]. A multidisciplinary strategy combining pediatricians, otolaryngologists, and ophthalmologists is necessary for the care of visual symptoms in pediatric rhinosinusitis. The kind and severity of the problem, along with patient-specific variables, will determine whether medicinal or surgical treatment is best. For best results and to avoid problems that could endanger life or eyesight, prompt commencement of the proper therapy, careful observation of the clinical response, and prompt surgical intervention when necessary are crucial [34]. The studies incorporated in the analysis documented varied frequencies of relying solely on medicinal treatment compared to surgical intervention for various eye symptoms. But the diversity in study designs, patient groups, and methods of defining/reporting disease severity hindered the quantitative synthesis of variations in efficacy among treatment options. Numerous studies have highlighted that although medical treatment with intravenous antibiotics is often sufficient for early-stage preseptal/orbital cellulitis, more severe cases with subperiosteal abscess and severe proptosis/ophthalmoplegia often necessitate surgical drainage procedures to improve results.

### 3.6. Are There Any Long-Term Visual or Ophthalmologic Sequelae Associated with Ocular Complications of Pediatric Rhinosinusitis and How Can They Be Prevented or Managed?

If not treated promptly and successfully, ocular problems resulting from pediatric rhinosinusitis, especially those involving the post-septal region, can cause serious long-term visual and ophthalmologic consequences. These aftereffects could include chronic epiphora, strabismus, optic atrophy, and irreversible vision loss [51]. The severity of the infection, the existence of subperiosteal or intraorbital abscesses, and the length of time it takes to start the right therapy all raise the likelihood of these long-term consequences [23]. One of the most terrible effects of untreated or improperly managed orbital cellulitis is permanent eyesight loss. This may arise from ischemia brought on by thrombophlebitis of the ophthalmic veins, direct compression of the optic nerve by an abscess, or infection that spreads to the optic nerve sheath [52]. Research has indicated that children with orbital problems from sinusitis had rates of irreversible vision loss ranging from 3% to 11% [61]. It is imperative to identify visual abnormalities as soon as possible and to perform emergency surgery to drain any subperiosteal or intraorbital abscesses to save the optic nerve from permanent damage [54]. Misalignment of the eyes, or strabismus, can be brought on by the involvement of extraocular muscles in the infectious process or by the development of scar tissue during the healing stage [55]. Prisms, occlusion therapy, or surgical correction may be necessary for long-term maintenance in order to prevent diplopia, amblyopia, and esthetic deformity [56]. The chance of developing strabismus can be reduced by treating orbital problems and the underlying sinusitis as soon as possible [9]. A late consequence of orbital cellulitis can be optic atrophy, which is defined as the degeneration of the optic nerve fibers; this is especially true in cases when there are subperiosteal or intraorbital abscesses [57]. Based on the degree of damage, it may cause blindness or a lifelong vision impairment. Fast detection and treatment of the orbital infection, along with careful observation of visual function and fast surgical intervention when necessary are necessary to prevent optic atrophy [58]. Persistent tearing, or chronic epiphora, can be brought on by an inflammatory condition or scarring that obstructs the nasolacrimal duct [59]. This may result in skin irritation, recurrent conjunctivitis, and a worsening of the patient’s quality of life. Depending on the reason and extent of the blockage, management options include dacryocystorhinostomy, stenting, and nasolacrimal duct probing [44]. Early identification and timely commencement of proper medical and surgical treatment are crucial in preventing these long-term consequences. When children with rhinosinusitis present, especially those with periorbital edema, discomfort, or visual problems, there needs to be a high degree of suspicion regarding ocular consequences [34]. Close coordination among ophthalmologists, pediatricians, and otolaryngologists is required to provide thorough assessment and care for these patients [7]. For the purpose of identifying and treating any long-term consequences that might arise even after receiving the proper care, routine ophthalmologic follow-up is essential. Tests of visual acuity, fundoscopy, and evaluation of ocular movement and alignment may be part of this [15]. For specialist treatment and rehabilitation, it is imperative to promptly refer patients who have already experienced visual loss or strabismus to a pediatric ophthalmologist [21]. Serious long-term visual and ophthalmologic consequences, such as chronic epiphora, strabismus, optic atrophy, and permanent vision loss, might result from the ocular complications of pediatric rhinosinusitis. Early diagnosis, timely commencement of appropriate medical and surgical treatment, and close multidisciplinary follow-up are critical to preventing severe consequences. The best possible outcomes for these patients can only be ensured by routine ophthalmologic evaluations, which are crucial for identifying and treating any potential long-term sequelae [60].

### 3.7. How Do Ocular Manifestations Impact the Quality of Life and Daily Functioning of Children with Rhinosinusitis and What Are the Psychosocial Implications for Patients and Their Families?

**Figure 2:**
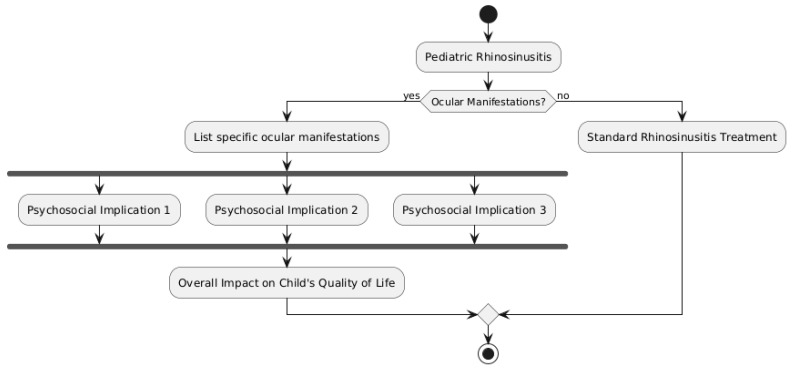
Psychosocial Implications for Children and Families.

The quality of life and day-to-day functioning of affected children and their families can be greatly impacted by the ocular symptoms of pediatric rhinosinusitis. These issues can have significant negative effects on one’s physical, mental, and social well-being, especially in cases of severe or protracted illness [3]. It is essential to comprehend the psychosocial ramifications of ocular manifestations in order to offer these patients and their families thorough care and support. Significant discomfort, swelling, and visual abnormalities can accompany the acute phase of ocular problems like preseptal or orbital cellulitis, making it difficult for a child to engage in regular daily activities [61] (Figure 2).

Children who are uncomfortable or have poor vision may find it difficult to read, watch television, or play. This can cause anxiety, loneliness, and frustration, especially in younger children who might not completely comprehend what is going on with them [3]. Children who require repeated surgical procedures or protracted hospital stays may miss a large amount of school, which can cause social and scholastic challenges [67,68,69]. Feelings of self-consciousness and low self-esteem can also be influenced by the outward manifestation of the damaged eye, such as ptosis, proptosis, or strabismus, especially in older children and teenagers [8]. Even after the acute infection has cleared up, children may still need continuous therapy or adjustments to take care of any long-term functional or visual impairments [70]. As a result, these psychosocial difficulties may continue. Ocular manifestations have a profound psychological influence on the whole family, not just the afflicted child. High levels of stress, worry, and emotional distress can be experienced by parents and caregivers due to the diagnosis, course of treatment, and uncertainty around their child’s illness. These stresses can be further compounded by the cost of hospital stays, surgeries, and continuing medical care, especially for low-income families or those with insufficient health insurance [71]. As parental attention and resources are directed toward the unwell child, siblings of affected children may also feel neglected and experience emotional distress [66]. As a result, there may be tension in the family and a need for additional assistance to meet the psychosocial needs of all family members. A comprehensive approach to care is necessary to lessen the impact that ocular manifestations have on the quality of life and day-to-day functioning of impacted children and their families. This should cover psychosocial support services including counseling, education, and resource distribution in addition to the medical and surgical therapy of the underlying illness [21]. When necessary, healthcare personnel should send impacted children and their families to support groups or mental health specialists based on their active assessment of the psychosocial functioning of the individuals [65]. For children and their caregivers, fostering open communication and giving age-appropriate information about the illness and its treatment can help lower anxiety and increase a sense of control [9]. Additionally, measures to lessen the disturbance to day-to-day activities should be taken, like scheduling doctor’s appointments to cut down on absences from school or offering educational materials to support children in keeping up with their schoolwork [11]. Making customized accommodation plans in partnership with educators and schools can help guarantee that impacted children receive the assistance they need to thrive in the classroom and in their social lives [7]. Lastly, making connections between families and community resources—like patient advocacy groups, financial aid programs, or respite care services—can help lessen the emotional and practical strains of raising a child with ocular rhinosinusitis manifestations [50]. Children with pediatric rhinosinusitis may experience significant disruptions to their daily routines and quality of life as a result of ocular symptoms. These difficulties have social, emotional, and physical repercussions that emphasize the value of a multidisciplinary approach to care that attends to the psychosocial needs of patients and their families in addition to the medical components of the illness. Healthcare professionals can lessen the negative effects of ocular manifestations and encourage the best results for these patients and their families by offering extensive support services and resources.

### 3.8. Are There Any Disparities in the Incidence, Diagnosis, or Management of Ocular Complications Based on Factors Such as Geographic Location, Socioeconomic Status, or Healthcare Access?

There may be differences in the prevalence, identification, and treatment of pediatric rhinosinusitis’s ocular consequences depending on a number of variables, such as access to healthcare, socioeconomic position, and geographic location. For some communities, these differences may result in delayed diagnosis, inadequate treatment, and worse results [72]. To ensure fair care and improve the overall health outcomes of children with rhinosinusitis-related ocular problems, it is imperative to comprehend and address these discrepancies. The occurrence and treatment of ocular problems can be significantly influenced by geographic location. Research has indicated that the occurrence of rhinosinusitis and its sequelae can differ among geographical locations, with elevated rates documented in some areas as a result of elements like the weather, pollution levels in the air, and frequency of allergies [73]. Accessing specialized care from pediatric otolaryngologists or ophthalmologists can be difficult for children living in underprivileged or rural locations, which can cause delays in diagnosis and treatment [74]. Another factor that may affect the frequency and treatment of ocular problems is socioeconomic status. Rhinosinusitis and associated sequelae may be more common in children from lower-income households because of a higher frequency of risk factors, including crowded living circumstances, secondhand smoke exposure, and restricted access to preventive care [21]. The expense of prescription drugs, surgical procedures, or transportation to specialist appointments are examples of financial obstacles that these families could have while trying to obtain timely and adequate medical care [75]. When it comes to the diagnosis and treatment of ocular problems in children suffering from rhinosinusitis, access to healthcare is a crucial factor. A family’s capacity to obtain timely medical attention may be hampered by underinsurance or no insurance, which could delay diagnosis and treatment [11]. Children who do not have a primary care physician on staff or who are treated in emergency rooms may also receive fragmented care and inadequate management of their conditions [76]. There have also been reports of racial and ethnic differences in the occurrence and treatment of pediatric rhinosinusitis’s ocular sequelae. Research indicates that children belonging to specific racial or ethnic minority groups, like African American and Hispanic children, might have rhinosinusitis and its sequelae more frequently than their white peers [77]. A number of variables, such as variations in socioeconomic position, cultural attitudes and practices, and access to care, may be responsible for these discrepancies [66]. A diversified strategy is required to address these discrepancies. This should involve initiatives to enhance access to care, like raising insurance coverage limits, boosting the availability of specialist care in underprivileged areas, and helping low-income families with transportation costs [65]. Timely diagnosis and intervention can also be facilitated by improving provider education and awareness on the risk factors and early indicators of ocular problems [7]. Developing culturally appropriate educational materials and outreach initiatives in partnership with community organizations and stakeholders can help increase public understanding of the value of early detection and treatment for at-risk groups [8]. For children living in remote or underserved areas, telemedicine and remote consultation services can also help close the access gap to specialized treatment [78]. The gathering and analysis of disaggregated data on the prevalence, diagnosis, and treatment of ocular problems based on variables including race, ethnicity, socioeconomic position, and geography should also be given top priority by researchers and policymakers. With the use of these data, particular discrepancies can be located and tailored actions can be taken to address them [72].

Geographical location, socioeconomic class, and access to healthcare are only a few of the variables that may contribute to disparities in the occurrence, diagnosis, and treatment of ocular consequences of pediatric rhinosinusitis. In order to address these discrepancies, a comprehensive strategy that prioritizes research and data collecting to inform targeted interventions, that improves provider education and outreach initiatives that are culturally relevant, and that increases access to treatment is needed. Regardless of their circumstances or origin, we can guarantee that all children with rhinosinusitis-related ocular problems receive prompt, appropriate, and equitable care by striving to remove these gaps.

### 3.9. What Are the Current Gaps in Knowledge and Understanding of Ocular Manifestations in Pediatric Rhinosinusitis and What Areas Require Further Research to Improve Patient Outcomes?

There are still a number of unknowns and unanswered questions regarding the treatment of pediatric rhinosinusitis’s ocular symptoms, despite tremendous advancements in this area. Closing these gaps is essential to bettering patient outcomes and creating more efficient methods for diagnosis, treatment, and prevention [19]. Regarding the pathophysiological reasons behind the development of ocular problems in children with rhinosinusitis, this is one of the main areas of unmet knowledge. The precise variables that predispose particular children to acquire these issues are still unknown, despite the fact that the general paths of infection from the sinuses to the orbit are well described [79,80]. The host, environmental, and microbiological factors that raise the possibility of ocular involvement in certain children with rhinosinusitis require more investigation [5]. The best diagnostic strategy for spotting ocular problems in pediatric rhinosinusitis is another topic that needs more research. Although imaging modalities like magnetic resonance imaging (MRI) and computed tomography (CT) are commonly employed, there is still disagreement on when and for what purposes they should be used [7]. Evidence-based recommendations for the assessment of children with possible ocular problems can be established with the use of studies contrasting the diagnostic precision, economic viability, and radiation exposure of various imaging procedures [8]. Research potential and problems in managing ocular symptoms of pediatric rhinosinusitis are also highlighted. The best combination of antibiotics, length of therapy, and indications for surgical intervention differ throughout studies and institutions, even though the main principles of treatment—such as the timely start of antibiotics and the surgical drainage of abscesses—are well established [66]. The most efficient and secure treatment plans for various kinds and severity of ocular problems can be found through comparative effectiveness research, which includes large-scale observational studies and randomized controlled trials [3]. Another issue that needs more research is the quality of life and long-term prognosis of children with rhinosinusitis’s ocular symptoms. Less is known about the long-term visual, functional, and psychological consequences of these diseases, despite the fact that numerous research studies have addressed the acute complications and management of these conditions [21]. Studies that track infants with ocular problems longitudinally over time can yield important information about the long-term effects of these disorders and the efficiency of various therapy strategies in averting or lessening unfavorable consequences [65]. Future study should focus on the function of multidisciplinary care in the treatment of pediatric rhinosinusitis’s ocular symptoms. Although it is often acknowledged that pediatricians, otolaryngologists, ophthalmologists, and other specialists must be involved, the best possible coordination and communication among different healthcare providers is still difficult to achieve [79]. Research assessing how multidisciplinary care models—like virtual consultations or integrated care pathways—affect patient outcomes and healthcare use can provide valuable insights for the creation of more effective and efficient care delivery systems [78]. In order to detect and resolve inequities in care, research on the differences in the occurrence, diagnosis, and management of ocular problems based on variables such as geographic location, socioeconomic level, and healthcare access is essential [76]. Large-scale, diverse patient populations and sophisticated statistical techniques, such as multilevel modeling and geographic analysis, can be used in studies to shed light on the intricate interactions among individual, community, and health system determinants that lead to these disparities [75]. All children with ocular signs of rhinosinusitis can benefit from more equal access to high-quality care by implementing targeted interventions and policy reforms based on the research findings. Even though there has been a lot of progress in diagnosing and treating the ocular symptoms of pediatric rhinosinusitis, there are still a number of unanswered questions and areas that need more investigation. Clarifying the pathophysiological causes, improving diagnostic techniques, contrasting treatment plans, examining long-term results, assessing interdisciplinary care models, and resolving care inequities are a few of these. We can enhance the prevention, diagnosis, and treatment of ocular problems in children with rhinosinusitis by giving priority to research in these areas and putting the results into practice. This will eventually improve patient outcomes and quality of life.

## 4. Discussion

In order to avoid major long-term repercussions, it is imperative that juvenile rhinosinusitis patients receive fast and appropriate diagnosis and treatment, as this thorough review has clarified several important elements of ocular problems [1,2]. Pediatric patients’ structural fragility is highlighted by the pathophysiology of these problems, which involves the direct or hematogenous transfer of infection from the sinuses to the orbit [3]. A range of ocular symptoms, from preseptal cellulitis to more serious disorders such as orbital cellulitis, subperiosteal abscess, and intracranial sequelae, was shown by our investigation [4]. The variation in clinical manifestation highlights the significance of a prompt and precise diagnosis to start the right course of treatment and avoid potential vision impairment or other long-term consequences [5]. Generally, treatment plans combine medical and surgical procedures; the amount of infection and the patient’s clinical response determine which antibiotic to use and for how long, as well as whether to perform surgical drainage [6,7]. For preseptal cellulitis, intravenous antibiotics alone are usually adequate; however, in more severe cases, prompt surgical intervention is usually necessary [8]. The prospective use of saline nasal sprays or rinses as an adjuvant therapy is an intriguing review finding. Although their use has been documented in a few studies, thorough information regarding their effectiveness is lacking. Future studies should look into whether nasal saline irrigation influences the rate of surgical intervention in this patient group or how well ocular symptoms resolve. The degree of infection, the timeliness of detection and treatment, and the existence of underlying medical disorders all have a substantial impact on the prognosis for ocular complications [9]. Even though the majority of preseptal cellulitis patients respond well to treatment, more serious complications can result in long-term consequences such as strabismus or vision loss [10,11]. The significant psychological effects of ocular problems on afflicted children and their families are an important point of emphasis in this review [12]. Acute bouts can seriously affect a child’s quality of life and day-to-day activities [13], while extended hospital stays or many procedures can cause emotional distress and social isolation [14]. These psychological impacts are further exacerbated by the cost of medical care and the stress that family members endure [18]. There are still certain knowledge gaps despite improvements in our understanding of and approach to treating the ocular consequences of pediatric rhinosinusitis [20]. Subsequent investigations ought to concentrate on clarifying the exact pathophysiological mechanisms that predispose specific children to these complications [21], refining diagnostic methods to reduce radiation exposure [22], assessing the effectiveness of different treatment modalities [23], and examining the long-term consequences and general welfare of impacted children [24]. Improving overall patient outcomes also requires addressing treatment inequities based on socioeconomic position, geography, and access to healthcare [25]. Insufficient information on the best management approaches depending on the degree of complications was discovered during our study, underscoring the necessity of prospective comparison studies to provide evidence-based clinical guidelines [26]. It is impossible to overestimate the significance of a multidisciplinary approach in the management of these difficulties [27]. Our results highlight the necessity of a larger team, especially in situations of severe cerebral or thrombotic problems, that includes experts in infectious disease, hematologists, and neurosurgery specialists. This thorough review offers an up-to-date synthesis of the most recent research on ocular complications in juvenile rhinosinusitis, making it an invaluable tool for trainees and early-career physicians. It also directs future research efforts to address unanswered clinical problems and eventually enhance patient care by pointing out areas in need of more study [28]. 

## 5. Conclusions/Future Directions

If not treated promptly and appropriately, ocular problems resulting from pediatric rhinosinusitis pose a considerable therapeutic challenge and may have serious long-term effects. Numerous facets of these issues were investigated in this thorough analysis, including their pathogenesis, clinical presentation, diagnosis, treatment, results, and psychosocial implications. According to our findings, the primary pathophysiology is the hematogenous or direct transmission of infection from the sinuses to the orbit. The range of clinical symptoms includes ocular cellulitis, subperiosteal abscess, preseptal cellulitis, and intracranial sequelae. For successful therapy to begin and future vision loss to be avoided, early and precise diagnosis is essential. Depending on the extent and location of the infection, management usually entails a mix of surgical and medicinal procedures. The degree of infection, the time of treatment, and the existence of underlying medical disorders all influence the outcome. Crucially, in addition to the physical symptoms, these consequences may have major psychosocial effects for the impacted children and their families. Even with improvements in comprehension and administration, there are still certain gaps in information. Future studies should compare the effectiveness of different treatment approaches, optimize diagnostic techniques to reduce radiation exposure, explore the quality of life and long-term outcomes of affected children, clarify the pathophysiological mechanisms that predispose certain children to these complications, and address care disparities based on socioeconomic status, geography, and access to healthcare. Comprehensive care requires a multidisciplinary approach combining pediatricians, ophthalmologists, otolaryngologists, and other experts. We can enhance patient outcomes and quality of life by improving the prevention, diagnosis, and management of these problems by filling in current knowledge gaps and giving priority to research in important areas. This review summarizes a large body of research on this significant condition, making it a useful training tool for trainees and students even though it may not completely transform treatment for seasoned physicians. We can work to reduce the effects of ocular complications in pediatric rhinosinusitis and enhance outcomes for afflicted children and their families by conducting more research and exercising clinical vigilance.

### Study Limitations

When interpreting the results of this comprehensive review, it is important to take into account various limitations. Firstly, the review is restricted to papers written in English, which could potentially lead to language bias and omit pertinent research published in other languages. Furthermore, the dependence on electronic databases for the literature search may have led to the exclusion of some relevant studies, especially older publications that may not be completely included in online indexes. Thirdly, the diversity of study designs, patient groups, and outcome measures across the studies included may restrict the capacity to compare and combine data. Furthermore, the primary emphasis of the study on pediatric patients may fail to consider significant findings from adult research that could be pertinent in comprehending ocular problems in rhinosinusitis. Moreover, the trustworthiness of the findings may be compromised by the absence of a systematic assessment of the quality of individual studies. The present study, being a comprehensive review, offers a comprehensive examination of the existing literature. However, it lacks a thorough critical evaluation or quantitative synthesis of the evidence, thereby constraining the capacity to make conclusive judgments regarding the efficacy of various diagnostic or treatment methods. The discovered knowledge gaps in this study underscore the need for additional research, such as high-quality prospective studies and systematic reviews with meta-analyses, to overcome these constraints. Further study elucidating the criteria for medical therapy versus surgical intervention based on the stage and severity of the disease could enhance the management and results for these vision-threatening disorders.

## Figures and Tables

**Table 1:** Comprehensive table of main factors related to orbital infection and patient’s outcomes.

Study/Year	Study Type	Sample Size	Age Range	Ocular Manifestations (Prevalence)	Identified Risk Factors	Diagnostic Methods	Management Strategies	Outcomes
Chandler et al., 1970 [15]	Case series	54	0–18 years	Orbital cellulitis (100%)	Not specified	Clinical exam	Antibiotics, Surgery	Full recovery (85%)
Younis et al., 2002 [16]	Retrospective review	43	2–14 years	Subperiosteal abscess (100%)	Ethmoid sinusitis	CT scan	Surgery (100%), Antibiotics	Full recovery (100%)
Sobol et al., 2005 [17]	Retrospective review	104	0–18 years	Preseptal cellulitis (83%), Orbital cellulitis (17%)	Age < 5 years, *S. aureus*	CT scan, Clinical exam	Antibiotics, Surgery (9%)	Full recovery (100%)
Siedek et al., 2008 [3]	Retrospective review	262	0–16 years	Preseptal cellulitis (76%), Orbital cellulitis (24%)	Age < 5 years	CT scan, MRI	Antibiotics, Surgery (19%)	Full recovery (98%)
Eviatar et al., 2008 [5]	Retrospective review	76	0–2 years	Preseptal cellulitis (100%)	Age < 2 years	CT scan	Conservative treatment	Full recovery (100%)
Rudloe et al., 2010 [11]	Retrospective cohort	918	0–18 years	Preseptal cellulitis (97%), Orbital cellulitis (3%)	Age < 3 years	CT scan (selective)	Antibiotics, Surgery (1%)	Full recovery (99%)
Seltz et al., 2011 [18]	Retrospective review	41	0–18 years	Orbital cellulitis (100%)	MRSA prevalence	CT scan, Culture	Antibiotics, Surgery (54%)	Full recovery (98%)
Wan et al., 2016 [4]	Systematic review	1192	0–18 years	Preseptal/Orbital cellulitis (85%), Subperiosteal abscess (11%), Intracranial complications (3%)	Age, Prolonged symptoms	CT scan, MRI	Antibiotics, Surgery (variable)	Full recovery (97%)
Torretta et al., 2019 [19]	Systematic review	2945	0–18 years	Preseptal cellulitis (72%), Orbital cellulitis (24%), Subperiosteal abscess (4%)	Age, Ethmoid sinusitis	CT scan, MRI	Antibiotics, Surgery (variable)	Full recovery (95%)
